# Differential physiological responses to environmental change promote woody shrub expansion

**DOI:** 10.1002/ece3.525

**Published:** 2013-03-13

**Authors:** Mary Heskel, Heather Greaves, Ari Kornfeld, Laura Gough, Owen K Atkin, Matthew H Turnbull, Gaius Shaver, Kevin L Griffin

**Affiliations:** 1 Department of Ecology, Evolution, and Environmental Biology, Columbia UniversityNew York, New York USA, 10027; 2 Department of Forest Ecosystems and Society, Oregon State UniversityCorvallis, Oregon, 97331, USA; 3 Department of Global Ecology, Carnegie Institution for ScienceStanford, California, 94305, USA; 4 School of Biological Sciences, University of CanterburyPrivate Bag 4800, Christchurch, New Zealand; 5 Department of Biology, University of Texas-ArlingtonArlington, Texas, 76019, USA; 6 Research School of Biology, The Australian National UniversityCanberra, ACT, 0200, Australia; 7 Ecosystems Center, Marine Biological LaboratoryWoods Hole, Massachusetts, 02543, USA; 8 Department of Earth and Environmental Sciences, Lamont-Doherty Earth Observatory, Columbia UniversityPalisades, New York USA, 10964-8000, USA

**Keywords:** *Betula nana nana*, carbon gain efficiency, *Eriophorum vaginatum*, Kok effect, photosynthesis, respiration, *Rubus chamaemorus*, tundra shrub encroachment

## Abstract

Direct and indirect effects of warming are increasingly modifying the carbon-rich vegetation and soils of the Arctic tundra, with important implications for the terrestrial carbon cycle. Understanding the biological and environmental influences on the processes that regulate foliar carbon cycling in tundra species is essential for predicting the future terrestrial carbon balance in this region. To determine the effect of climate change impacts on gas exchange in tundra, we quantified foliar photosynthesis (*A*_net_), respiration in the dark and light (*R*_D_ and *R*_L_, determined using the Kok method), photorespiration (PR), carbon gain efficiency (CGE, the ratio of photosynthetic CO_2_ uptake to total CO_2_ exchange of photosynthesis, PR, and respiration), and leaf traits of three dominant species – *Betula nana*, a woody shrub; *Eriophorum vaginatum*, a graminoid; and *Rubus chamaemorus*, a forb – grown under long-term warming and fertilization treatments since 1989 at Toolik Lake, Alaska. Under warming, *B. nana* exhibited the highest rates of *A*_net_ and strongest light inhibition of respiration, increasing CGE nearly 50% compared with leaves grown in ambient conditions, which corresponded to a 52% increase in relative abundance. Gas exchange did not shift under fertilization in *B. nana* despite increases in leaf N and P and near-complete dominance at the community scale, suggesting a morphological rather than physiological response. *Rubus chamaemorus*, exhibited minimal shifts in foliar gas exchange, and responded similarly to *B. nana* under treatment conditions. By contrast, *E. vaginatum*, did not significantly alter its gas exchange physiology under treatments and exhibited dramatic decreases in relative cover (warming: −19.7%; fertilization: −79.7%; warming with fertilization: −91.1%). Our findings suggest a foliar physiological advantage in the woody shrub *B. nana* that is further mediated by warming and increased soil nutrient availability, which may facilitate shrub expansion and in turn alter the terrestrial carbon cycle in future tundra environments.

## Introduction

The vegetation and soils of northern latitudes account for approximately 33–50% of the global terrestrial carbon pool, and impacts associated with climate change are altering the carbon balance of this system (Gorham 1991; Oechel et al. 1993; Ping et al. 2008; Tarnocai et al. 2009). Arctic warming has resulted in multiple cascading ecological and environmental effects that individually and collectively threaten profound impacts on the terrestrial carbon cycle (Post et al. 2009; Shaver et al. 1992). Of these, one of the drivers of change with the greatest potential impact is the rapid conversion of low, mostly nonwoody tundra vegetation to taller vegetation dominated by deciduous shrubs (Chapin et al. 2005), and several recent reports suggest that this conversion is happening in many tundra regions (Sturm et al. 2001; Tape et al. 2006). As both warming and shrub encroachment increasingly modify tundra, it is essential to understand how abundant species assimilate, store, and respire carbon, necessitating accurate measures of independent foliar carbon cycle components in this quickly changing landscape.

Shrubs can alter tussock tundra physically and biologically on multiple scales, with many impacts attributable to their higher canopy stature. The taller canopies of shrubs increase snow depth, which leads to greater insulation during winter months, increasing soil microbial activity, and in turn soil nutrient availability during spring snowmelt (Schimel et al. 2004; Sturm et al. 2005b; Weintraub and Schimel 2005). Taller, denser canopies also intercept more solar radiation in the summertime, leading to decreases in soil thaw and cooler soil temperatures in the summer (Chapin et al. 1995; Blok et al. 2011; Shaver et al. 2013). Furthermore, taller canopies increase surface roughness of the tundra landscape, altering its energy balance (Chapin et al. 2005; Liston et al. 2002), and may alter timing of snowmelt (Pomeroy et al. 2006), which has implications for landscape albedo (Sturm et al. 2005a) and species' phenology (Van Wijk and Williams 2003). Like shrubs, graminoid species also show measurable responses to climate change impacts on the tundra (Chapin et al. 1995) Evidence suggests that these responses may be mediated by, and arguably are a direct result of, the growth and proliferation of shrubs, such as *Betula nana*, measured in our study, at the individual, community, and ecosystem level (Shaver et al. 2001; Wookey et al. 2009). *Eriophorum vaginatum*, a common tundra graminoid, is particularly well studied in previous leaf, whole plant, and community studies due to its high abundance, and its ecology, under current and potential future conditions is well documented (Wein and Bliss 1974; Shaver and Chapin 1986; Chapin and Shaver 1996). A nondominant but abundant species, *Rubus chamaemorus*, also considered in this study, fills a different ecological niche as a low-lying, shade-tolerant deciduous forb may also play a more important role in future tundra composition and production.

Long-term field experiments underscore the effects of environmental change on community and ecosystem properties in Arctic tundra (Chapin et al. 1995; Hobbie and Chapin 1998; Elmendorf et al. 2012), and a detailed look at foliar fluxes in common tundra species may clarify these relationships. Understanding the biological and environmental influences on the processes that regulate the foliar processes in tundra species is essential for predicting the future terrestrial carbon balance in this region, as only at the leaf level can the relevant photosynthetic and respiratory parameters may be measured. Given the inherent differences among the common tundra species measured in this study – a woody, deciduous, clonal, ectomycorrhizal shrub; a tillering, nonmycorrhizal tussock sedge; and a deciduous, shade-loving, arbuscular mycorrhizal forb – it is likely leaf-level gas exchange will differ among species according to metabolic demand, and potentially shift differentially under warming and nutrient availability. Knowledge of these intra- and interspecies differences in photosynthesis and respiration will yield more accurate estimations of larger scale carbon fluxes under current and future warming.

The balance of photosynthetic and respiratory rates controls foliar carbon fluxes, and here we define a novel metric of carbon gain efficiency (CGE) as a ratio of the rate of carbon assimilated through photosynthesis to the total carbon assimilated through photosynthesis and released via photorespiration (PR) and respiration. A greater CGE may indicate lower respiratory costs for tissue maintenance and ion transport or enhanced photosynthetic capacity (Lambers et al. 2008). Conversely, a decreased CGE may result in decreased overall carbon storage. The enzyme-mediated pathways of photosynthesis and respiration respond to changes in ambient air and soil temperature and soil nutrient availability associated with climate change in the Arctic tundra (Shaver et al. 1992; Wookey et al. 2009), and this adaptive shift may promote the migration and establishment of shrubs through increases in CGE of shrubs relative to nonshrub species.

Plants in the Arctic tundra experience near complete or complete 24-hour photoperiods during the growing season, and this extreme light environment should be considered when evaluating foliar gas exchange, as multiple leaf-level carbon fluxes are sensitive to light. Thus, accurate evaluation of foliar carbon exchange and CGE in tundra plants requires estimates of both respiration in the light (*R*_L_) and PR in addition to photosynthesis (*A*_net_) and dark respiration (*R*_D_). Although important to quantify as a baseline maximum rate of potential mitochondrial respiration, incorporation of only *R*_D_ would not reflect in vivo rates of mitochondrial carbon efflux in arctic plants, as foliar respiration is reduced in light compared with respiration in the dark due to alterations in multiple enzymatic pathways associated with the tri-carboxylic acid cycle (Kok 1948; Sharp et al. 1984; Budde and Randall 1990; Atkin et al. 1997; Hoefnagel et al. 1998; Tcherkez et al. 2005). *R*_L_ can be sensitive to multiple environmental factors including atmospheric CO_2_ concentration, irradiance, temperature, and water availability (Leegood et al. 1995; Wang et al. 2001; Shapiro et al. 2004; Hurry et al. 2005; Pärnik et al. 2007; Tcherkez et al. 2008; Ayub et al. 2011; Griffin and Turnbull 2013). Currently, little is known about the degree of biochemical inhibition of respiration in the light in arctic plants, or if they adapted to limit or eliminate this inhibition in an environment with constant daylight during the growing season. Here, we also consider PR, another environmentally sensitive foliar CO_2_ efflux (Collatz 1977; Brooks and Farquhar 1985; Leegood and Edwards 2004), as the neglect of these fluxes could lead to a potentially significant miscalculation of respiration, gross photosynthesis, and the resulting ecosystem carbon balance. A predictive and mechanistic understanding of ecosystem carbon flux in arctic tundra requires knowledge of how both processes response to current and predicted environmental change.

We argue that differences observed at the community and ecosystem scale associated with woody shrub expansion may be consistent with, and in part attributable to, tundra plant species' foliar carbon cycling and metabolic efficiency. We hypothesized that shrubs of the Alaskan Arctic tundra would have a higher CGE at the leaf level than other abundant tundra species, and that this difference could represent a physiological advantage that is consistent with increasing shrub density under Arctic climate change. To test this under current and predicted environmental scenarios, we quantified photosynthesis, respiration in the dark and light, PR, and related leaf traits in three dominant tundra species representing three different functional groups under ambient and experimental conditions that simulated impacts associated with climate change, specifically warming, and increased soil nutrient availability. A related objective was to quantify the light inhibition of respiration in these species and to examine the importance of this phenomenon in foliar carbon fluxes and metabolism. Our study represents one of the first reports of respiration in the light and PR, two environmentally sensitive, potentially substantial, yet often neglected C fluxes, in these dominant Arctic tundra species under long-term warming and fertilization. The new detailed data on these mechanistic processes will allow for a better understanding on the carbon efficiency in these species, and their potential relationships with known community level shifts in Arctic vegetation under simulated global change.

## Materials and Methods

### Field site and species

The study took place during the peak growing season from mid-June through mid-July 2009 at the Arctic Long Term Ecological Research (LTER) field site near Toolik Lake (68°38′N, 149°36′W) on Alaska's North Slope, located 254 km north of the Arctic Circle. All leaves were fully expanded and from the upper canopy, and were sampled from experimental plots in moist acidic tundra (MAT) established and maintained by the LTER since 1989 (similar to an older experiment described by Chapin and Shaver 1985). The MAT site consists of four randomized blocks of 5 × 20-m treatment plots separated by a 1-m buffer arrayed two-by-two on a slightly sloped, poorly drained hillside. Treatments manipulate the ambient environment to reflect predicted impacts of climate change on tussock tundra, including increased soil and air temperature using greenhouses (GH), increased nitrogen and phosphorus availability using fertilizers (NP), and a combined treatment of warming and fertilization (GHNP). Wood-framed greenhouses, covered with transparent 0.15-mm plastic sheeting, passively increase air temperature by approximately 5°C during the growing season, while insignificantly affecting light intensity, humidity, and thaw depth (Gough and Hobbie 2003). In the NP plots, 10 g/m^2^ of granular NH_4_NO_3_-N and 5 g/m^2^ of granular P_2_O_5_-P are applied each year in early June after snowmelt. In the GHNP treatment plots, tundra vegetation is enclosed by the same greenhouses as in the GH plots and treated with the same fertilizers, in the same amounts, as in the NP plots.

The focal species for our study are common and abundant at the MAT site: *Eriophorum vaginatum* L. (“cottongrass”), the tussock-forming sedge that gives “tussock tundra” its name and is widely distributed over much of Alaska's North Slope, northern Canada, and northern Eurasia; *Betula nana nana* L. (“dwarf birch”), a deciduous woody shrub that is also abundant and often dominant over much of the entire Arctic region; and *Rubus chamaemorus* (“cloudberry”), a widespread and often abundant herbaceous perennial forb. In this study on leaf properties of these species, care was taken to ensure a sampling of only fully expanded leaves of a similar size and age.

### Foliar gas exchange

CO_2_ fluxes of photosynthesis and respiration were measured using an infrared gas analyzer (IRGA; LI-6400XT Portable Photosynthesis System, LI-COR, Lincoln, NE). All measurements were taken on clipped leaves collected from the long-term MAT treatment plots, recut under water in the field, and transported in water to the laboratory. Preliminary tests on these species showed no difference in rates of gas exchange and stomatal conductance between field-measured and lab-measured leaves (K. L. Griffin, unpubl. data), as has been shown previously in other species (Mitchell et al. 1999; Turnbull et al. 2003). This sampling technique was required to provide a greater degree of temperature control, maximize the number of replicates, and minimize time between replicates over the short growing season and during potentially rapidly changing environmental conditions in the field. For each *E. vaginatum* sample, we collected approximately 10 leaves from a single tussock in order to provide sufficient leaf area in the leaf chamber (∼6 cm^2^). *Betula nana* leaves, which were smaller than the 6 cm^2^ cuvette area, were measured for leaf area after IRGA measurements and gas exchange values were corrected to that area for analysis. Individual leaves of *R. chamaemorus* covered the entire leaf cuvette for gas exchange measurements. Two replicate measurements per species were made on leaves collected from each treatment block.

Prior to measurement, leaves were enclosed in the cuvette at high light conditions to acclimate. CO_2_ assimilation was measured under ambient (400 ppm) CO_2_ concentration under 26 levels of photosynthetically active radiation (PAR): 1500, 1200, 800, 400, 200, 100, and every 5 PAR between 100 and 0 μmol/m^2^ per sec. This range of light fully encompasses the light environment experienced by the three species in their growth environment (Heskel et al. 2012). After 10 minutes in darkness, it was assumed that no photosynthesis or PR was taking place, and all CO_2_ flux could be attributed to mitochondrial respiration in the dark (*R*_D_). All measurements were taken at a relative humidity of approximately 40–60%, and potential diffusion in and out of the cuvette was accounted for, as was diffusion through the gasket, according to corrections presented in the LiCor 6400 Instructional Manual. Average leaf vapor pressure deficit was 0.717 ± 0.081 (SE) across all treatments. To account for the influences of leaf temperature on gas exchange variables, cuvette block temperature was set to 20°C for all measurements, which is representative of temperatures experienced during the growing season (Heskel et al. 2012). Maximal light-saturated net photosynthetic rate (*A*_net_) was estimated by fitting the data to a rectangular hyperbolic function (Excel Solver, Microsoft, Redmond, WA). PR, which represents the CO_2_ flux release associated with the oxygenation of Rubisco at saturating light, was calculated according to equations presented by von Caemmerer and Farquhar (1981), as described in Ayub et al. (2011). Using values of photosynthesis, PR, and respiration in the light (*R*_L,_ see Kok effect below), CGE was calculated (CGE = *A*_net_/[*A*_net_ + *R*_L_ + PR]) to estimate the proportional carbon gain per carbon exchanged. Values of photosynthesis and respiration are expressed on an area, mass, and nitrogen basis.

### Quantifying respiration in the light using the Kok effect

To estimate respiration in the light, we used the Kok method, which is convenient for field measurements and can be used under ambient atmospheric conditions. This method is based on the observation that the quantum yield of photosynthesis usually decreases abruptly above a certain level of light intensity – often near the light compensation point, where carbon flux is zero (Kok 1948). This leads to a noticeable nonlinearity, or “bend,” in the otherwise linear lower range of the light-response curve, which is interpreted as the saturation point of light inhibition of respiration (*I*_RL_, explained in detail in Shapiro et al. 2004). As respiration is assumed to be constant above this point, an extrapolation to 0 μmol/m^2^ per sec PAR of the linear portion of the curve above this point is assumed to give the rate of respiration in the light (Fig. S1). Here, the irradiance range we used to calculate *R*_L_ spanned from 25 to 90 μmol/m^2^ per sec. The percent inhibition of light respiration is calculated from the ratio of the difference between dark and light respiration rates to the rate of dark respiration: % *I*_RL_ = ([*R*_D_ – *R*_L_]/*R*_D_) × 100.

The Kok method assumes the CO_2_ assimilation rate responds only to light, and thus corrections must be made to account for changes in internal CO_2_ (*c*_i_). As rates of photosynthesis slow under decreasing light intensity, CO_2_ tends to accumulate within the leaf, increasing *c*_i_, which in turn affect the shape of the light curve by decreasing rates of PR at lower light levels. We corrected all measurements to a constant *c*_i_ to account for these effects, according to Kirschbaum and Farquhar (1987), as described in Ayub et al. (2011), to achieve a more accurate extrapolation of *R*_L_.

### Physical leaf traits and foliar nutrients

All leaf samples used for gas exchange measurements were measured for leaf area using a rotating-belt leaf area meter (LI-3100C Area Meter, LI-COR, Lincoln, NE). Samples were then dried in an oven at 60°C for a minimum of 2 days before mass was determined. After transport to Columbia University in New York, all samples were ground, weighed, and packaged for elemental analysis of [CHN] (2400 Series II, Perkin-Elmer, Boston, MA). Remaining ground leaf samples were bulked by replicate block (*n* = 4) and sent to the North Carolina State University Environmental and Agricultural Testing Service (Raleigh, NC) for analysis by wet digestion to determine total phosphorus concentration.

### Relative species abundance

The cover of mosses, lichens, and all vascular plant species was measured in eight 1 × 1-m^2^ plots in each block in mid-July 2007 by visual estimation as described by Gough and Hobbie (2003). The relative cover was calculated by dividing the individual species' cover by the total plant cover for each measured quadrat (Gough and Hobbie 2003). Major functional group- and species-level community shifts correlated with warming, fertilization, and warming with fertilization treatments occurred prior to 2007, so these data should represent the vegetation that occurred in these plots in 2009 with reasonable confidence (L. Gough, unpubl. data).

### Statistical analyses

The effects of environmental treatment on gas exchange variables and leaf characteristics were analyzed using a three-way analysis of variance (ANOVA) in R (v2.7.0, The R Foundation for Statistical Computing), assigning species, fertilization, and warming as explanatory variables. Block effects were not found to be significant for the variables measured. Tukey's Honestly Significant Difference test was used for multiple comparisons of means. Community data were analyzed using a two-way multivariate ANOVA (MANOVA) with *B. nana*, *E. vaginatum*, *R. chamaemorus*, and “other” (an aggregate of all other species found in the plots but not considered in this study) as dependent variables, and warming and fertilization as factors. A Wilks' lambda test statistic was used to determine significance. Differences were considered significant if *P* < 0.05.

## Results

### Species-mediated variation in leaf chemistry and traits

Leaf N was greatest in the deciduous species, *B. nana* and *R. chamaemorus*, while *E. vaginatum* exhibited the lowest concentrations (main effect of species: *P* < 0.001, *F*_2, 94_ = 14.414). A significant interaction between warming and fertilization was present among all species (*P* < 0.01, *F*_1, 95_ = 11.072), where warming dampened the magnitude of increase under fertilization. Within individual species, leaf N of *E. vaginatum* and *B. nana* increased in NP plots (Table 1). In leaf P, a significant interaction effect between warming and fertilization was observed (*P* < 0.01, *F*_1, 47_ = 7.549), driven by differences in *B. nana* and *R. chamaemorus*, where the combined treatment values were lower than NP plots alone. Similar to leaf N, the lowest concentrations were observed in *E. vaginatum* (main effect of species: *P* < 0.001, *F*_2, 46_ = 12.679). No significant warming effect independent of fertilization was detected upon analysis. In terms of foliar C:N, the interaction between warming and fertilization (*P* < 0.001, *F*_1, 95_ = 14.159) resulted in greater values between species. Foliar C:N varied among species (main effect of species: *P* < 0.001, *F*_2, 94_ = 22.234), with greatest values observed in leaves of *E. vaginatum*. Among species, fertilization altered foliar C:N, mainly through higher measures of N in leaves grown under fertilization (Table 1, *P* < 0.001, *F*_1, 95_ = 33.403). Foliar N:P did not vary significantly among species, although was predictably influenced by fertilization treatment (*P* < 0.001, *F*_1, 47_ = 25.099). This trend was also apparent when considering individual species, where N:P was considerably lower under fertilized growth conditions compared to the control leaves (Table 1).

**Table 1:** Foliar element concentrations and ratios of the three study species grown under control and treatment conditions

		N (mmol/g)	P (μmol/g)	C:N	N:P
*Betula nana*	CT	1.92 ± 0.03^a^	98.5 ± 5.5	17.78 ± 0.33^a^	19.58 ± 0.79^a^
	GH	1.94 ± 0.09^a^	106.5 ± 3.2	17.35 ± 0.57^a^	18.30 ± 1.20^a^
	NP	2.51 ± 0.16^b^	200.2 ± 7.4^bc^	13.23 ± 0.63^b^	12.58 ± 0.65^b^
	GHNP	2.03 ± 0.10^a^	158.2 ± 2.9^b^	17.14 ± 0.85^a^	12.82 ± 0.74^b^
*Eriophorum vaginatum*	CT	1.69 ± 0.09^a^	85.3 ± 2.3^a^	20.08 ± 1.11^a^	19.85 ± 1.06^a^
	GH	1.62 ± 0.05^a^	92.3 ± 1.5^a^	20.35 ± 0.54^a^	17.64 ± 0.38^ab^
	NP	2.10 ± 0.05^b^	126.8 ± 3.7^b^	15.73 ± 0.33^b^	16.78 ± 0.79^ab^
	GHNP	1.85 ± 0.05^a^	143.7 ± 6.5^bc^	17.75 ± 0.54^a^	12.97 ± 0.69^b^
*Rubus chamaemorus*	CT	2.11 ± 0.11^a^	138.8 ± 11.0^a^	15.51 ± 0.84^a^	15.79 ± 1.23^a^
	GH	2.06 ± 0.08^a^	118.5 ± 3.8^a^	15.49 ± 0.55^a^	17.53 ± 0.93^a^
	NP	2.44 ± 0.12^a^	200.2 ± 1.2^b^	13.27 ± 0.76^a^	12.24 ± 0.66^a^
	GHNP	2.05 ± 0.11^a^	126.4 ± 22.2^a^	15.79 ± 0.86^a^	17.92 ± 1.98^a^

N and P values represent mmol/g of leaf area, and N:P is a ratio of those values, however, C:N represents a ratio of the percent of dry leaf matter for both elements. *N* = 7–9 for N, C:N, and *n* = 4 for P and N:P. Means ± SE are presented, and statistical significance (*P* < 0.05) is denoted alphabetically within species.

For all species, SLA was significantly affected by warming (*P* < 0.05, *F*_1, 95_ = 5.457), mainly driven by *R. chamaemorus*, of which SLA increased with elevated growth temperature (species-treatment interaction effect: *P* <0.01, Fig. S2). Similarly, fertilization did not increase SLA, except in the case of *R. chamaemorus* (Fig. S2).

### Species and warming drive differences in carbon exchange

Mean values of net photosynthesis (*A*_net_) were greatest in *B. nana* and lowest in *E. vaginatum* (Table 2), and species' effects were highly significant on an area, mass, or N basis (all *P* < 0.001, Table S1). Considering all replicates, neither fertilization nor warming treatments significantly altered photosynthesis. However, leaves of *E. vaginatum* exhibited a general decline in rates under warming in both the nonfertilized and fertilized plots. Similarly, across all replicates, PR in *E. vaginatum* was significantly lower than in *B. nana* and *R. chamaemorus* (Table 2). Between species, PR calculated in leaves grown under the combined GHNP treatment was elevated compared to the individual treatments, NP and GH alone (Table 2). Under warming, PR of *E. vaginatum* was lower than in leaves of both *B. nana* (*P* < 0.05) and *R. chamaemorus* (*P* = 0.197), although when combined with fertilization this effect was not found.

**Table 2:** Foliar photosynthesis, photorespiration for the three species under control and treatments (*n* = 7–10)

	*A*_net_ (μmol/m^2^ per sec)				PR (μmol/m^2^ per sec)			
		
	CT^1^	GH^1^	NP^1^	GHNP^1^	CT^1^	GH^1^	NP^1^	GHNP^2^
*Betula nana*^A^	16.99 ± 1.50^b^	16.95 ± 1.38^b^	13.92 ± 1.63^a^	13.22 ± 1.64^a^	1.46 ± 0.16^a^	1.28 ± 0.12^b^	0.93 ± 0.11^a^	1.33 ± 0.20^a^
*Eriophorum vaginatum*^B^	10.02 ± 1.49^a^	6.20 ± 0.99^a^	11.75 ± 1.38^a^	9.67 ± 1.16^a^	0.99 ± 0.16^a^	0.51 ± 0.08 ^a^	0.93 ± 0.12^a^	1.68 ± 0.45^a^
*Rubus chamaemorus*^C^	12.24 ± 0.80^ab^	11.65 ± 0.91^ab^	13.14 ± 1.22^a^	13.98 ± 1.05^a^	1.02 ± 0.09^a^	1.08 ± 0.10^ab^	1.24 ± 0.11^a^	1.59 ± 0.18^a^

Values are expressed on an area basis and represent means ± SE; significance was tested for species, warming, and fertilization effect by three-way ANOVA. Species that do not share a capital letter, growth conditions that do not share a number, and individual means that do not share a lowercase letter are significantly different (*P* > 0.05).

**Figure 1:**
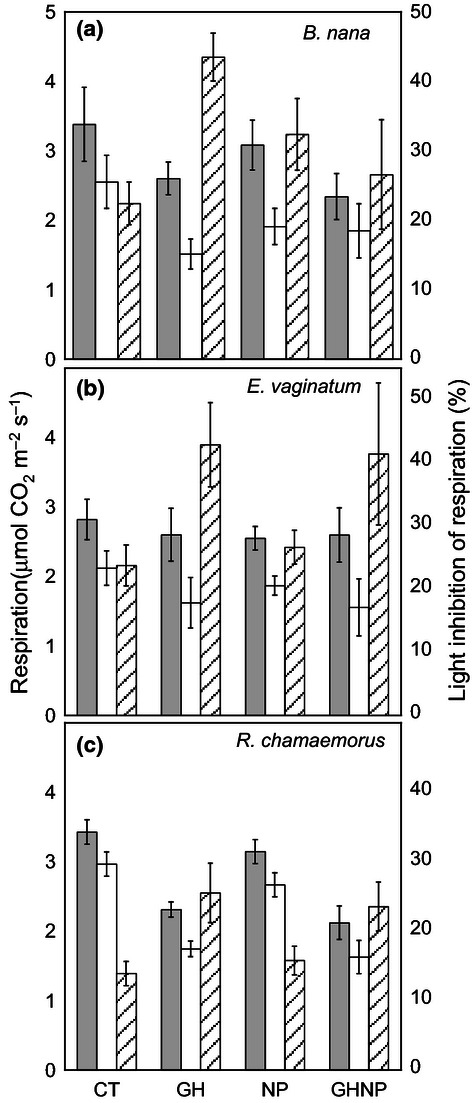
Respiration in the dark (shaded) and light (unshaded) and the corresponding inhibition of respiration by light (diagonally striped) in *Betula nana* (a), *Eriophorum vaginatum* (b), and *Rubus chamaemorus* (c). Values represent means ± SE, *n* = 8 for all variables. Alphabetical notation was not used for clarity.

Dark respiration (*R*_D_) did not vary significantly among species when expressed by area, although highest rates were reported in *B. nana* when expressed on a mass- and N-basis. Among species, warming decreased area-based *R*_D_ (−21.7 ± 6.8%, Table S1), and mass-based *R*_D_ (−21.4 ± 11.1%, Table S1). Within species, no significant treatment effects were observed in *R*_D_ (Fig. 1)_._ Light significantly inhibited respiration (*P* < 0.0001) across all replicates, and the percentage inhibition of respiration (% *I*_RL_) ranged widely (Fig. 1), with a mean of 27 ± 2% across species and treatments. Rates of respiration in the light (*R*_L_), like *R*_D_, were lowest in *E. vaginatum* (Fig. 1) and varied among species on an area- (*P* < 0.05), mass- (*P* < 0.001), and N basis (*P* < 0.001, Table S1). Warming further reduced rates of respiration among species when expressed by area: −36.9 ± 8.3% (*P* < 0.001), mass: −22.3 ± 9.1% (*P* < 0.001), and N: −20.1 ± 8.1%, (*P* < 0.05). Fertilization treatment had a similar, although less significant effect, with mean values of *R*_L_ lower than control values across species, although only by area (*P* < 0.05) and N (*P* < 0.01). No significant interactions between warming and fertilization were found.

**Figure 2:**
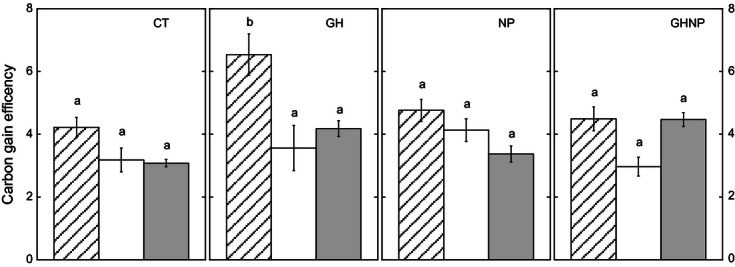
Carbon gain efficiency of *Betula nana* (diagonal stripe), *Eriophorum vaginatum* (unshaded), *Rubus chamaemorus* (lightly shaded) under control conditions and the three treatments (*n* = 8). Values represent means ± SE; alphabetical notation indicates significance between treatments within a species at *P* < 0.05.

CGE values were greatest in *B. nana*, and significantly lower in both *R. chamaemorus* (*P* < 0.05) and *E. vaginatum* (*P* < 0.001, Fig. 2). Across species, both warmed and fertilized growth conditions minimally influenced CGE, with effects of 3.4 ± 1.7% and 3.5 ± 1.8%, respectively. Within an individual species, only rates of CGE in *B. nana* exhibited significant elevation: warming more than doubled CGE compared to control values (*P* < 0.05, Fig. 2). Furthermore, under warming, CGE of *B. nana* was significantly greater than the other two species (both *P* < 0.01). There were no clear significant correlations of mass-based fluxes with N, P, or N:P (Fig. S3), although *B. nana* and *R. chamaemorus* showed higher rates of gas exchange and CGE than *E. vaginatum* per unit N or P.

### Shrub dominance and tussock vulnerability under manipulated environmental conditions

At the community scale, fertilized and warming treatments exhibited a significant interaction effect on relative species abundance considering all measured species, and among individual species, only *B. nana* had a significant interaction effect (Table 3).

**Table 3:** Results from a two-way MANOVA on the relative abundance of the three focal species, with fertilization (NP) and warming (GH) as factors

Source	Overall			*Betula nana*			*Rubus chamaemorus*		*Eriophorum vaginatum*	
			
	df	*F*	*P*	df^1^	*F*	*P*	*F*	*P*	*F*	*P*
NP	3, 91	204.76	***	1, 93	299.95	***	13.36	***	166.22	***
GH	3, 91	4.03	**	1, 93		*ns*		*ns*	10.19	***
NP × GH	3, 91	9.45	***	1, 93	11.55	***		*ns*		*ns*

MANOVA, multivariate analysis of variance; df, degree of freedom; *ns*, not significant. *F*-values were not reported for nonsignificant results.

1 Degrees of freedom for *B. nana F*-tests were the same for the other two species.

Stars represent significance as follows: *P < 0.05, **P < 0.01, ***P < 0.001.

**Figure 3:**
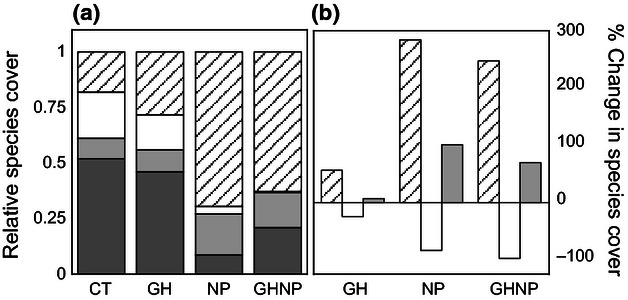
Relative species cover across treatments (a) and percent change in species cover relative to control plot measurements (b); *Betula nana* (diagonal stripe), *Eriophorum vaginatum* (unshaded), *Rubus chamaemorus* (lightly shaded), and all other measured species were combined (darkly shaded).

Relative species abundance, both overall and considering individual species, was significantly influenced by fertilization (Table 3). Warming affected the relative species abundance overall, but only affected *E. vaginatum* individually, significantly decreasing its abundance (*P* < 0.005, Fig. 3). Mean relative abundance values of *B. nana* increased under warming, further under fertilization and the combined treatment (all *P* < 0.05), compared to control values, whereas *R. chamaemorus* increased in cover under fertilization and *E. vaginatum* significantly decreased under both warming and fertilization treatments (Fig. 3). Overall, *B. nana* responded most in terms of expanding cover under long-term treatments, becoming the dominant species in the NP and GHNP plots, while in those same plots, *E. vaginatum* was most negatively affected.

## Discussion

This study presents new insights into leaf-level carbon exchange in the light in three common and abundant Arctic species, under current ambient and predicted future environmental conditions, with special attention to mechanistic responses in light, specifically mitochondrial respiration in the light and PR, given the nightless environment of the tundra growing season. Incorporating these novel measurements to quantify CGE provides relevant measures of carbon loss in Arctic plants, and considered together, our findings suggest a foliar physiological advantage in the leaves of woody shrub *B. nana* over the graminoid *E. vaginatum* and forb *R. chamaemorus* that may enable community-scale dominance and be further mediated by warming and increased soil N and P availability.

### Species-level physiological differences under current conditions

Our results indicate strong species-driven differences in leaf-level gas exchange and underlying leaf nutrient composition under ambient environmental conditions. We found higher rates of CO_2_ assimilation in leaves of *B. nana* than the other species, mirroring differences in net photosynthesis measured in nearby plots nearly three decades ago (Chapin and Shaver 1996). Rates of respiration in the light and dark were lowest in *E. vaginatum* and highest in *B. nana* and *R. chamaemorus* (Fig. 1) potentially reflecting potential differences in energy demand required for new leaf growth and development. Similarly, when compared to *E. vaginatum*, both *B. nana* and *R. chamaemorus* exhibited greater leaf N and P, which corresponded to greater net photosynthetic and respiratory rates (Fig. S3). *R*_L_ and PR are important variables to quantify as autotrophic respiration may account for nearly 50% of ecosystem respiration in tundra system (Hicks-Pries et al. 2013). The inclusion of these two parameters allowed the first known calculation of foliar CGE in these species. *Betula nana* exhibited the highest rate of CGE in ambient growth conditions due to the higher proportion of CO_2_ assimilation to CO_2_ loss through PR and *R*_L_, and it is possible much of that carbon that would be allocated toward woody stem growth and expansion (Bret-Harte et al. 2002). At the community scale, our results also show similar species composition in control plots to previous measurements (Shaver and Chapin 1986; Shaver et al. 2001), indicating long-term consistency in composition under ambient environmental conditions. This consistency exhibited in the control plots provide a stable background against which experimental manipulations of key environmental changes can be measured.

### Physiological responses to warming and fertilization promote shrub expansion

Environmental treatments simulating future impacts of climate change on the Alaskan arctic tundra facilitated a restructuring of the community. The woody shrub, *B. nana*, increased in abundance disproportionally under elevated air temperature, and to a stronger degree under increased soil N and P availability and in a combination of the two treatments, ultimately replacing the graminoid *E. vaginatum* and leading to a stark alteration of relative species composition (Fig. 3). Despite similar trends at the community scale, the foliar physiology within and across species responded differently to these environmental treatments both on a mass and area basis, suggesting important distinctions in the physiological and morphological mechanisms that may facilitate shrub expansion in a future climate.

*Betula nana* exhibited the greatest rates of CGE under warming (Fig. 2), due to the compounded effect of a high rate of *A*_net_ and lower rates of PR and *R*_L_ compared to control plants. *E. vaginatum* and *R. chamaemorus* exhibited general increases in CGE, mainly due to decreases in PR and *R*_L_ in *E. vaginatum*, and decreases in *R*_L_ in *R. chamaemorus*. *A*_net_ showed no increase under warming, as reported previously in Arctic tundra vegetation at the leaf (Chapin and Shaver 1996) and ecosystem scale (Welker et al. 2004; Biasi et al. 2008; Huemmrich et al. 2010), potentially indicating a long-term loss of stimulatory effect. However, for *E. vaginatum*, *A*_net_ in leaves grown under warming was lower than in leaves grown in ambient conditions, suggesting this species is operating beyond its temperature optimum. It is also possible that in vivo leaf temperature may be altered by increased cover density in a shrubbier environment, potentially lowering rates through an acclimation to the cooling effect of shading in a taller canopy. The influence of warming on photosynthesis is likely to depend on the duration and degree of the warming treatment, and is shown to vary across region and microclimate (Oberbauer et al. 2007; Elmendorf et al. 2012). All species in this study exhibited a general trend of greater inhibition of respiration in the light under warming, which contrasts with results from a previous study (Atkin et al. 2006). The lower mean *R*_D_ and *R*_L_ values under warming across the three species also suggest long-term thermal acclimation of respiration (Atkin and Tjoelker 2003). Taken as a whole, foliar gas exchange is modified under warmer temperatures, and allows for significantly greater C accumulation in *B. nana*, creating a competitive physiological advantage compared to the nonwoody species. When considering the community, this may lead to more relative cover of woody shrubs under warming, mirroring the trend already established in Arctic tundra (Chapin et al. 1995; Tape et al. 2006; Walker et al. 2006). In addition to altering leaf physiology in *B. nana*, warming can stimulate belowground growth and N uptake (Hobbie and Chapin 1998; Gough and Hobbie 2003; Sullivan et al. 2007). Furthermore, warming may alter components of the associated ectomycorrhizal community that promote nutrient acquisition (Deslippe et al. 2011) and belowground carbon transfer in *B. nana* (Deslippe and Simard 2011). Conversely, the decrease in relative cover in the historically abundant graminoid *E. vaginatum* may be in part related to leaf-level physiology due to its tendency to decrease *A*_net_ under warming (Table 2), its less efficient photosynthetic N-use efficiency compared to *B. nana*, as well as herbivory (Gough et al. 2012) and interspecies competition for resources such as light and soil nutrients in a shrubbier canopy (Fetcher 1985).

In contrast to warming, long-term fertilization did not impact CGE in any of the study species. *Betula nana* and *R. chamaemorus*, both mycorrhizal-forming, had higher leaf N and P concentrations than *E. vaginatum*, though this did not translate to enhanced rates of carbon exchange (Fig. S3). An earlier study including these three species found a similar lack of stimulatory effect of fertilization on maximal photosynthesis despite increases in shoot growth (Bigger and Oechel 1982). Previous experiments reported elevated photosynthesis under the same fertilization treatment after ∼3 years (Chapin and Shaver 1996), although decreases were reported by Bret-Harte et al. (2001) after ∼8 years, which may suggest a temporal duration of fertilization effect, and potentially foliar N-acclimation after a relatively short time period. Values of N:P found in this study do not indicate photosynthetic limitation by either N or P (Tessier and Raynal 2003), and a lack of a clear photosynthesis-N relationship (Fig. S3) in these species, also observed along water tracks (Griffin and Turnbull 2013) suggests the allocation of N toward other cellular processes, such as nitrate assimilation and storage or cell wall material (Onoda et al. 2004; Takashima et al. 2004), or wood production (Bret-Harte et al. 2002, 2001).

While foliar carbon fluxes exhibited little positive response to greater N and P availability, significant changes were observed at the community scale (Fig. 3A). Fertilization, under both ambient and warm temperatures enabled the dominance of *B. nana* and the near disappearance of *E. vaginatum* from the treatment plots. *Rubus chamaemorus*, although not as responsive as *B. nana*, also expanded its presence under elevated N and P (Fig. 3B). This expansion of the woody shrub *B. nana* is noted in previous studies in the tundra of Arctic Alaska (Bret-Harte et al. 2002; Chapin et al. 1995; Mack et al. 2004; Shaver et al. 2001; Walker et al. 2006) and appears to be a widespread phenomenon. In contrast to the foliar physiological response observed under warming, this may indicate a morphological adaptive strategy under fertilization, where shoot initiation and expansion, and thus total leaf area, increases, but the physiology of those leaves is relatively unaltered. This implies a strong relationship between total foliar N, leaf area index, and gross primary productivity (Van Wijk et al. 2005; Williams and Rastetter 1999), although this may be limited by self-shading or decreased photosynthetic N-use efficiency (Street et al. 2007). In addition, considering the similar rates of CGE in the species under fertilization, the concurrent dominance of *B. nana* at the community level suggests that belowground interactions may be responsible for driving plant composition changes. This is supported by earlier studies in which a marked decline in the fine root production of *E. vaginatum* under long-term N and P fertilization (Sullivan et al. 2007), and increases in ectomycorrhizal fungi associated with *B. nana* (Clemmensen et al. 2006) were associated with greater aboveground dominance of *B. nana*. Furthermore, although long-term elevated soil N and P may enhance total NPP and aboveground C and N pools, over time belowground C losses from soil may outpace gains from gross primary productivity (Chapin et al. 1995; Mack et al. 2004).

The combined growth treatment of warming with fertilization revealed decoupled impacts on leaf physiology. For all study species, leaf N and P declined under the combined treatment compared to fertilization alone (Table 1), suggesting, in the case of *B. nana*, a dilution of tissue N and P due to greater shoot initiation and expansion under the combined treatment. Foliar respiration in the coupled treatment reflected similar trends as seen under warming alone, suggesting thermal acclimation for all three species, and an elevation of the inhibition of respiration in the nonshrub species. The effect of the coupled treatment did not lead to either enhanced photosynthesis or CGE, in concordance with previous measurements at the ecosystem scale (Chapin and Shaver 1996; Boelman et al. 2005). However, the combined treatments still strongly altered community composition with comparable decreases in *E. vaginatum* and increases in *R. chamaemorus* and *B. nana* as observed under fertilization alone. Together, these data confirm N and P to be a greater limitation to shrub growth and expansion, and in turn a greater threat to tundra biodiversity, than temperature, and underscore the importance of multifactor global change experiments to understand terrestrial carbon cycling (Templer and Reinmann 2011).

### Implications and conclusions

When considering the carbon reservoir of the Arctic tundra and its future fate, it is important to include estimates of leaf-level physiological responses of tundra species and how they may change under future predicted conditions. To that end, we present the first ecologically meaningful estimates of respiration to consider the known inhibition of respiration caused by light from the well-studied, long-term global change experiment at Toolik Lake, Alaska. By combining estimates of *R*_L_ with carefully measured photosynthetic rates and calculated rates of PR, we present for the first time estimates of CGE in arctic tundra species. While the difference in environmental treatments result in a similar phenomenological response, this study demonstrates that a number of different physiological mechanisms are responsible. Our results indicate a shift in foliar gas exchange physiology that may provide an advantage to the dominant woody shrub species *B. nana*, facilitating its expansion into the historically tussock-dominated tundra. Furthermore, we describe the possibility of a more morphological/developmental strategy on the part of *B. nana* that may mediate aboveground expansion when soil N and P are not limiting. Prior to our experiments, it was unknown to what degree these two mechanisms contributed to the observed increase in shrub growth, and only through the quantification of the relevant physiological measurements could these relationships be determined. Therefore, despite similar plot-level responses, the underlying mechanisms vary, providing critical information for modeling, and requiring further study to fully understand the long-term ecosystem consequences. Also, the synergistic impacts of both warming and increased N and P show interactions that do not reflect an additive effect, which suggest the need for more research on multiple factors on these processes.

The use of physiological measurements suitable to quantifying the mechanistic responses in the unique Arctic environment of continuous daylight provides new insights into the regulation of carbon in this important ecosystem. The incorporation of these findings into vegetation models will more accurately reflect the mechanistic underpinnings of the ecosystem response to global change and improve our predictive understanding. Future modeling studies can benefit from integrating these foliar physiological processes and their temperature and seasonal responses at the canopy and ecosystem level, as they may reveal new insights into carbon allocation. Ultimately, knowing more about species' individual and community growth responses under environmental change and the corresponding relative carbon fluxes will enable more accurate estimates of ecosystem carbon storage. Our results suggest that different functional strategies under the individual treatments may allow for the continued encroachment and expansion of woody shrubs into the Arctic tundra as temperatures warm in this region, and this has significant implications for both biodiversity and carbon storage.

## Supporting Information

**Figure S1.** Example of light-response curve at low photosynthetically active radiation (PAR) displaying the Kok effect. Unshaded points above the bend in the slope extrapolate to *R*_L_ on the *y*-axis. Shaded points below the breakpoint decrease at a faster rate and terminate at *R*_D_ where PAR = 0 μmol/m^2^ per sec.

**Figure S2.** Specific leaf area of the three study species grown under treatment conditions (*n* = 8). Values presented are means ± SE; alphabetic notation denotes significance between treatments within a species at *P* < 0.05.

**Figure S3.** Mass-based rates of photosynthesis, respiration in the light, and carbon gain efficiency of *Betula nana* (circle), *Eriophorum vaginatum* (triangle), *Rubus chamaemorus* (square) plotted against mass-based N, P, and N:P. Values represent means under treatments ± SE.

**Table S1.** Analysis of variance (ANOVA) results for gas exchange variables, comparing the three study species under all growth conditions. Variables analyzed include area-, mass-, and nitrogen-based photosynthesis (*A*_net_), dark respiration (*R*_D_), respiration in the light (*R*_L_), degree of respiratory inhibition by light (% *I*_RL_), photorespiration (PR), and carbon gain efficiency (CGE). For all variables measured or calculated, *n* = 7–10 for all species treatment combinations. Stars represent significance as follows: **P* < 0.05, ***P* < 0.01, ****P* < 0.001.
