# Supplementary material for: Differential physiological responses to environmental change promote woody shrub expansion
Source: Ecol Evol. 2013 Mar 13;3(5):1149–62. doi: 10.1002/ece3.525 (PMC3678471; doi:10.1002/ece3.525)
Supplement: Supplementary file 1 [file ece30003-1149-SD1.docx]

**Supplemental Tables and Figures**

**Table S1.** ANOVA results for gas exchange variables, comparing the three study species under all growth conditions. Variables analysed include area-, mass-, and nitrogen-based photosynthesis (*A*_net_), dark respiration (*R*_D_), respiration in the light (*R*_L_), degree of respiratory inhibition by light (% *I*_RL_), photorespiration (PR), and carbon gain efficiency (CGE). For all variables measured or calculated, *n* = 7-10 for all species-treatment combinations*.* Stars represent significance as follows: * *p* < 0.05, ** *p* < 0.01, *** *p* < 0.001.

|  |  | Species | |  | NP | |  | Warming | |  | NP x Warming | |
| --- | --- | --- | --- | --- | --- | --- | --- | --- | --- | --- | --- | --- |
|  | *d.f.* | *F* | *P* | *d.f.* | *F* | *P* | *d.f.* | *F* | *P* | *d.f.* | *F* | *P* |
| *A*_net-area_ | 2, 94 | 18.08 | *** | 1, 95 | 0.300 | *ns* | 1, 95 | 1.992 | *ns* | 1, 95 | 0.301 | *ns* |
| *R*_D-area_ | 2, 94 | 6.80 | *ns* | 1, 95 | 1.528 | *ns* | 1, 95 | 16.815 | *** | 1, 95 | 0.086 | *ns* |
| *R*_L-area_ | 2, 94 | 3.18 | * | 1, 95 | 1.381 | *ns* | 1, 95 | 23.046 | *** | 1, 95 | 1.967 | *ns* |
| *A*_net-mass_ | 2, 94 | 86.51 | *** | 1, 95 | 0.531 | *ns* | 1, 95 | 0.526 | *ns* | 1, 95 | 1.113 | *ns* |
| *R*_D-mass_ | 2, 94 | 63.31 | ** | 1, 95 | 0.245 | *ns* | 1, 95 | 10.159 | ** | 1, 95 | 1.244 | *ns* |
| *R*_L-mass_ | 2, 94 | 41.88 | *** | 1, 95 | 0.208 | *ns* | 1, 95 | 11.801 | *** | 1, 95 | 0.001 | *ns* |
| *A*_net-N_ | 2, 94 | 82.03 | *** | 1, 95 | 1.005 | *ns* | 1, 95 | 23.871 | * | 1, 95 | 0.111 | *ns* |
| *R*_D-N_ | 2, 94 | 53.95 | *** | 1, 95 | 6.857 | * | 1, 95 | 0.273 | *ns* | 1, 95 | 0.172 | *ns* |
| *R*_L-N_ | 2, 94 | 33.25 | *** | 1, 95 | 5.906 | * | 1, 95 | 3.325 | < 0.10 | 1, 95 | 2.779 | < 0.10 |
| % *I*_RL_ | 2, 94 | 8.78 | *** | 1, 95 | 0.231 | *ns* | 1, 95 | 16.067 | *** | 1, 95 | 4.699 | * |
| PR | 2, 94 | 5.40 | ** | 1, 95 | 3.357 | < 0.10 | 1, 95 | 1.539 | *ns* | 1, 95 | 12.330 | *** |
| CGE | 2, 94 | 11.80 | *** | 1, 95 | 1.706 | *ns* | 1, 95 | 1.974 | *ns* | 1, 95 | 1.506 | *ns* |

**Figure S1.** Example light-response curve at low PAR displaying the Kok effect. Unshaded points above the bend in the slope extrapolate to *R*_L_ on the y-axis. Shaded points below the breakpoint decrease at a faster rate and terminate at *R*_D_ where PAR = 0 μmol m^-2^ s^-1^.

**Figure S2.** Specific leaf area of the three study species grown under treatment conditions (*n* = 8). Values presented are means ± SE; alphabetic notation denotes significance between treatments within a species at *p* < 0.05.

**Figure S3.** Mass-based rates of photosynthesis, respiration in the light, and carbon gain efficiency of *B. nana* (circle), *E. vaginatum* (triangle), *R. chamaemorus* (square) plotted against mass-based N, P, and N:P. Values represent means under treatments ± SE.
